# Intrarenal Dopaminergic System Is Dysregulated in SS-*Resp18^mutant^* Rats

**DOI:** 10.3390/biomedicines11010111

**Published:** 2023-01-01

**Authors:** Usman M. Ashraf, Ealla Atari, Fawaz Alasmari, Harshal Waghulde, Vikash Kumar, Youssef Sari, Sonia M. Najjar, Pedro A. Jose, Sivarajan Kumarasamy

**Affiliations:** 1 Department of Physiology and Pharmacology, University of Toledo College of Medicine and Life Sciences, Toledo, OH 43614, USA; 2 Department of Pharmacology and Experimental Therapeutics, University of Toledo College of Pharmacy & Pharmaceutical Sciences, Toledo, OH 43614, USA; 3 Department of Physiology, Medical College of Wisconsin, Milwaukee, WI 53226, USA; 4 Department of Biomedical Sciences, Heritage College of Osteopathic Medicine, Ohio University, Athens, OH 45701, USA; 5 Diabetes Institute, Heritage College of Osteopathic Medicine, Ohio University, Athens, OH 45701, USA; 6 Department of Medicine, Division of Kidney Diseases & Hypertension, The George Washington University School of Medicine & Health Sciences, Washington, DC 20052, USA; 7 Department of Pharmacology and Physiology, The George Washington University School of Medicine & Health Sciences, Washington, DC 20052, USA

**Keywords:** Resp18, kidney, blood pressure, dopamine, Dahl salt-sensitive rats

## Abstract

The genetic and molecular basis of developing high blood pressure and renal disease are not well known. *Resp18^mutant^* Dahl salt-sensitive (SS-*Resp18^mutant^*) rats fed a 2% NaCl diet for six weeks have high blood pressure, increased renal fibrosis, and decreased mean survival time. Impairment of the dopaminergic system also leads to hypertension that involves renal and non-renal mechanisms. Deletion of any of the five dopamine receptors may lead to salt-sensitive hypertension. Therefore, we investigated the interaction between *Resp18* and renal dopamine in SS-*Resp18^mutant^* and Dahl salt-sensitive (SS) rats. We found that SS-*Resp18^mutant^* rats had vascular dysfunction, as evidenced by a decrease in vasorelaxation in response to sodium nitroprusside. The pressure–natriuresis curve in SS-*Resp18^mutant^* rats was shifted down and to the right of SS rats. SS-*Resp18^mutant^* rats had decreased glomerular filtration rate and dopamine receptor subtypes, D1R and D5R. Renal dopamine levels were decreased, but urinary dopamine levels were increased, which may be the consequence of increased renal dopamine production, followed by secretion into the tubular lumen. The increased renal dopamine production in SS-*Resp18^mutant^* rats in vivo was substantiated by the increased dopamine production in renal proximal tubule cells treated with L-DOPA. Overall, our study provides evidence that targeted disruption of the *Resp18* locus in the SS rat dysregulates the renal dopaminergic system.

## 1. Introduction

Hypertension is a multifactorial polygenic disease that is associated with a high risk for cardiovascular disease and a major risk factor for stroke and renal disease [1,2,3]. It is the leading cause of chronic kidney disease (CKD) that currently affects 15% of the US population [4]. The onset and progression of hypertension and associated renal disease are affected by genetic and environmental factors, such as an increase in dietary salt intake, smoking, and alcohol consumption [5,6,7,8,9]. However, the genetic and molecular basis of increased risk for developing high blood pressure and renal disease are not well known. It is generally accepted that 30–50% of blood pressure abnormalities can be attributed to genetic factors [10,11]. Thus, identifying genes/genetic loci that contribute to high blood pressure is fundamental in understanding this complex disease.

The Dahl salt-sensitive (SS) rat is one of the most extensively studied models of salt-induced hypertension and renal injury [5,12,13,14,15,16]. Substitution mapping studies conducted in SS rats led to the identification of genetic loci responsible for salt-induced hypertension and renal disease [5,16,17,18]. One such study led to the identification of regulated endocrine-specific protein-18 (*Resp18*) as a candidate gene for blood pressure regulation [17]. *Resp18* was first identified by screening a rat neuro-intermediate pituitary *cDNA* library for transcripts, the expression of which is regulated by dopaminergic agents in parallel with the endogenous prohormone pro-opiomelanocortin [19]. Subsequently, deep RNA-sequencing analysis detected *Resp18* gene expression in renal proximal tubule cells [20].

*Resp18*, as a candidate gene for hypertension, was validated by generating an SS-*Resp18^mutant^* rat on SS rat genetic background using a zinc-finger nuclease approach, targeting exon 3 of the *Resp18* gene [21]. This leads to a seven-base frameshift deletion of bases in the SS-*Resp18^mutant^* rats and introduces a premature stop codon in mutant rats [21]. We reported that a high-salt diet (2% NaCl) increased both systolic and diastolic blood pressures in SS-*Resp18^mutant^* rats to a greater extent than SS rats, which was associated with a greater increase in renal fibrosis and urinary protein excretion [21]. Dopaminergic agonists decrease the expression of *Resp18,* whereas dopaminergic antagonists increase its expression, suggesting a molecular link between *Resp18* and dopamine [19,22]. Apart from its role as a neurotransmitter, dopamine mediates other essential physiological functions, including the regulation of blood pressure, water and electrolyte balance, and renal function [23,24,25]. Dopamine can be produced in the kidney, independently of the central nervous system [24,25,26,27]. L-3-4-dihydroxyphenylalanine (L-DOPA) from the circulation or glomerular filtrate is reabsorbed in renal proximal tubule cells, where it is converted into dopamine by DOPA decarboxylase. Dopamine is then secreted into the renal tubular lumen and basolateral space, where it inhibits ion transport [23,24,25,26,27].

We have shown that SS-*Resp18^mutant^* rats maintained on a high-salt diet had higher blood pressure and urinary protein excretion and lower mean survival time than wild-type SS rats [21]. Given that *Resp18* is expressed in renal proximal tubule cells and its expression changes in response to dopaminergic agents (D_2_-like receptor antagonist haloperidol increases and the D_2_-like receptor agonist bromocriptine decreases *Resp18* expression), it is possible that *Resp18* plays a crucial role in the regulation of salt-induced increase in blood pressure and consequently, renal injury. The present study tests this hypothesis by examining whether targeted disruption of the *Resp18* gene in SS rats increases blood pressure and causes impairment of renal function by abolishing/disrupting the renal protective effect of the renal dopaminergic system.

## 2. Materials and Methods

### 2.1. Animals

SS-*Resp18^mutant^* rats were generated on the SS rat genetic background by using the zinc-finger nuclease method, as previously detailed [21,28]. Male Dahl salt-sensitive/Mcw (SS) and SS-*Resp18^mutant^* rats were bred, housed, and raised on a low-salt diet (0.3% NaCl; Harlan Teklad diet 7034) until six weeks of age before switching them to a high-salt (2% NaCl; Harlan Teklad diet 94217) diet for the remainder of the experimental protocol. All animals were kept on a 12:12-h light-dark cycle in a climate-controlled room. Rat chow and water were provided ad libitum. All animal research protocols were approved by the Institutional Animal Care and Use Committee of the University of Toledo, in accordance with the National Institutes of Health Guide for the Care and Use of Laboratory Animals.

### 2.2. Food and Water Intake

After six weeks on a high-salt diet, SS and SS-*Resp18^mutant^* rats were housed individually in a comprehensive laboratory animal monitoring system (CLAMS) for four days. Food and water intakes of each rat were recorded in real-time, as routinely done.

### 2.3. Vascular Myograph

After six weeks on a high-salt diet, SS and SS-*Resp18^mutant^* rats were euthanized by the CO_2_ inhalation method. The second- and third-order mesenteric arteries were dissected and placed in cold Krebs–Henseleit solution (KHS), pH 7.4. The segments, 2 mm in length, were mounted in wire myograph chambers (Danish Myo Tech, model 610 M; JP-Trading I/S). For isometric tension recording, two steel wires were introduced through the lumen of the mounted arteries. The arteries were allowed to equilibrate in KHS for 15 min. The arterial diameters were determined after stretching to their optimal lumen diameter based on the internal circumference/wall tension. The vessels were then washed again with KHS and allowed to equilibrate for 20 min. The concentration–response curve was first measured for acetylcholine (ACh) (10^−9^ M to 10^−4.5^ M). Thereafter, the arteries were washed and allowed to equilibrate in KHS for 20 min before the concentration–response curve for sodium nitroprusside (SNP) (10^−9^ M to 10^−4.5^ M) was assessed.

### 2.4. Glomerular Filtration Rate in Conscious Rats

Glomerular filtration rate (GFR) was measured in conscious SS and SS-*Resp18^mutant^* rats via the transcutaneous clearance of fluorescein–isothiocyanate (FITC)–sinistrin, using a NIC-Kidney device (Mannheim Pharma & Diagnostics GmbH, Mannheim, Germany) [29,30,31]. The rats were anesthetized for ~10 min (2% *v*/*v* isoflurane). Thereafter, the device was turned on by connecting it to a rechargeable lithium battery and then attached to the back of the rat using a double-sided adhesive tape; the device was protected with one layer of adhesive gauze tape. After recording the baseline period for ~2–5 min, FITC-sinistrin (5 mg/100 g dissolved in physiological saline solution) was injected into the tail vein. Each rat was placed into an individual cage to minimize the risk of probe dislodgement. After a 2 h recording period, the device was carefully removed, and the data was analyzed using NIC-Kidney device partner software (MPDlab v1.0, Mannheim Pharma & Diagnostics, GmbH). All rats had ad libitum access to food and water except during the 2 h GFR measurement period.

### 2.5. Immunohistochemistry of the Kidney

The kidneys were dissected from 12-week-old SS and SS-*Resp18^mutant^* rats maintained on a high-salt diet starting at six weeks of age. The dissected kidneys were fixed in 10% formalin and embedded in paraffin. The slides were deparaffinized in xylene washes and rehydrated with graded series of ethanol. The kidney sections were then incubated in PBS with 3% H_2_O_2_ for 10 min to inactivate endogenous peroxidase. The slides were washed for 5 min in PBST (PBS + 1% Tween 20) and blocked with 3% bovine serum albumin in PBST (blocking buffer) for 2 h at 4 °C. Rabbit anti-CD68 (1:100; Santa Cruz, Dallas, TX, USA; SC-70760) was diluted in blocking buffer and incubated at 4 °C overnight. The slides were washed three times for 30 min in PBST, and biotinylated goat anti-rabbit secondary antibody (1:500, Abcam, Cambridge, UK; ab64256) was used for development with avidin-biotinylated HRP complex (Vectastain ABC Elite kit; PK-6100; Vector Laboratories, Newark, CA, USA), followed by counterstaining with hematoxylin and mounted for image capture. For primary antibody control, the tissues were incubated with a blocking buffer without the primary antibody. Once processed and prepared for imaging, the kidney slides were viewed, and images were captured with a Nikon Ni-E motorized upright microscope equipped with DS-QiMc camera and NIS-Element software. Twenty fields (0.56 mm^2^ each) were randomly selected from each renal cortex and outer medulla. The numbers of immunolabelled cells were counted manually or by an automated counting method.

### 2.6. Measurement of Dopamine

Urinary dopamine concentrations were measured by the Neurochemistry Core at the Vanderbilt University’s Center for Molecular Neuroscience Research. Dopamine concentrations in the renal cortex and cell culture medium were quantified using the HPLC-EC method [32,33]. In brief, perchloric acid (HClO_4_) (0.25 N) was used for the lysis and sonication of renal cortex samples. Subsequently, the samples were centrifuged at 14,000× *g* for 20 min at 4 °C. The supernatants were collected and filtered through a 0.22 μm filter, and the pellets were saved for protein quantification. The filtered samples were then injected onto a C18 column (3.2 × 150 mm, 3 μm particle size, Thermo Scientific, Waltham, MA, USA). The reagents (54.3 mM sodium phosphate, 0.215 mM octyl sodium sulphate, 0.32 mM citric acid, and 11% methanol (pH ~4.4)) were mixed to prepare the mobile phase. For the detection of dopamine in the renal cortex and cell culture medium, the CoulArray coulometric array detector (model 5600 A, ESA, Inc., Paris, France) was used, and the dopamine peaks were seen on the chromatograms of the CoulArray software. The external dopamine standard was used to determine the area under the curve of standard peaks using different concentrations. Based on the established standard curve, dopamine concentrations in the renal cortex of both groups were measured. Total protein was measured to normalize the dopamine concentration in the renal cortex relative to the amount of protein in each sample.

### 2.7. Immunoblotting

At twelve weeks of age, after six weeks on a high-salt diet, the SS and SS-*Resp18^mutant^* rats were euthanized, and the kidneys immediately snap-frozen. Total protein from the kidney was isolated using TPER reagent (Thermofisher, USA), containing protease and phosphatase inhibitor cocktail (Pierce, Appleton, WI, USA). Protein concentrations in the lysates were measured using the BCA colorimetric method (Thermo Fisher, USA). From each sample, 40 μg of protein was used for Western blot analysis. The following primary antibodies were used: D1R (EMD Millipore, Burlington, MA, USA, #MAB5290), D5R (EMD Millipore, #MAB5292), and GAPDH (Cell Signaling Technology, Danvers, MA, USA, #14C10).

### 2.8. Sodium Measurement

At twelve weeks of age, after six weeks on a high-salt diet, the SS and SS-*Resp18^mutant^* rats were individually placed in metabolic cages for 24 h urine collection [21]. The rats were provided free access to drinking water. Urine sodium was measured using the enzymatic sodium test kit (DZ114b-K) per the manufacturer’s instructions.

### 2.9. Isolation and Culture of Renal Proximal Tubule Cells and Dopamine Release Assay

Renal proximal tubule cells were isolated from renal cortical slices obtained from SS and SS-*Resp18^mutant^* rats [34,35] and placed in primary culture media. Dopamine released from the cultures of renal proximal tubule cells isolated from SS and SS-*Resp18^mutant^* rat was assayed, as reported [32,33,36]. In brief, renal proximal tubule cell monolayers, seeded into six-well plates, were washed and pre-incubated with and without reserpine, for 20 min at 37 °C before L-DOPA was added into the wells. The monoamine oxidase inhibitor pargyline (10 µM) and the catechol-O-methyltransferase inhibitor tolcapone (1 µM) were added into the cell culture dish 20 min before the experiment to prevent the enzymatic degradation of dopamine. After 20 min incubation, the renal proximal tubule cells were incubated with L-DOPA (75 µM) in HBSS for 2 h at 37 °C; the concentration of dopamine in the incubation media reached the maximum with 75 μM L-DOPA [36]. The media were collected to measure dopamine concentrations at 0, 30, 60, and 120 min. The inhibitors were present during the entire period of time. Twenty-five µL of 0.25 N HClO_4_ were added to one ml of cell supernatant and stored at −80 °C. The amount of dopamine in the cell supernatants was measured by HPLC-EC [32,33,36].

### 2.10. RNA Isolation and Quantitative Real-Time-PCR

Total RNA was isolated from renal proximal tubule cells using Trizol Reagent (Invitrogen, Eugene, OR, USA), according to the manufacturer’s protocol. RNA purity and concentration were determined by NanoDrop One (Thermofisher). One µg of DNase-treated total RNA was used for first-strand complementary DNA synthesis using M-MLV reverse transcriptase (Promega, Madison, WI, USA), per the manufacturer’s protocol. Quantitative PCR was performed in the Quantstudio 5 Real-Time PCR machine (Life Technologies, Carlsbad, CA, USA), using Power SYBR Green PCR master mix (Invitrogen) and gene-specific primers for *Resp18* (*Resp18*-RT-F; ATCCAGCGAAGATGCAGAGT, *Resp18*-RT-R; ACCATCGTGGGCATTTATGT). The gene expression data were normalized to *Gapdh* (*Gapdh*-RT-F; CAAGATGGTGAAGGTCCGTGTG, and *Gapdh*-RT-R; AGAGCCTGTGTCCATACTTTG). Gene expressions were calculated by the delta–delta Ct method and expressed as fold-change relative to SS rats [21].

### 2.11. Statistical Analysis

Data are presented as mean ± standard error of the mean (SEM). Data were analyzed by *t*-test or two-way ANOVA (Sidak test), as appropriate, with a *p*-value of <0.05, a threshold for statistical significance.

## 3. Results

### 3.1. SS-Resp18^mutant^ Rats Have Vascular Dysfunction and Reduced Glomerular Filtration Rate (GFR)

**Figure 1:**
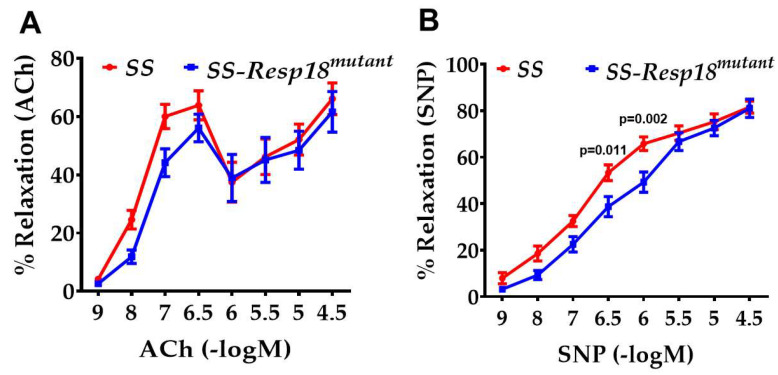
SS-*Resp18^mutant^* rats have vascular dysfunction after six weeks of a high-salt diet: Concentration–response curve to (**A**) acetylcholine (ACh) and (**B**) sodium nitroprusside (SNP) in mesenteric arteries isolated from SS and SS-*Resp18^mutant^* rats (*n* = 4–6/group). Values are mean ± SEM. *p* = 0.011, *p* = 0.002 vs. SS-*Resp18^mutant^*, two-way ANOVA (Sidak test).

To determine whether the increase in blood pressure observed in SS-*Resp18^mutant^* rats [21] was associated with vascular dysfunction, vasoreactivity was measured in second- and third-order mesenteric arteries mounted on a vascular bath [5]. Endothelium-dependent vasorelaxation to acetylcholine (ACh) was assessed by adding increasing concentrations of ACh (10^−9^ M to 10^−4.5^ M) to the bathing medium. ACh-induced vasorelaxation tended to be decreased in SS-*Resp18^mutant^* compared with SS control rats but did not reach statistical significance (Figure 1A). Similar to ACh, endothelium-independent vasorelaxation was assessed by adding increasing concentrations of SNP (10^−9^ M to 10^−4.5^ M) to the bathing medium. Endothelium-independent vasorelaxation induced by SNP was significantly decreased in SS-*Resp18^mutant^* rats compared with SS rats (Figure 1B).

**Figure 2:**
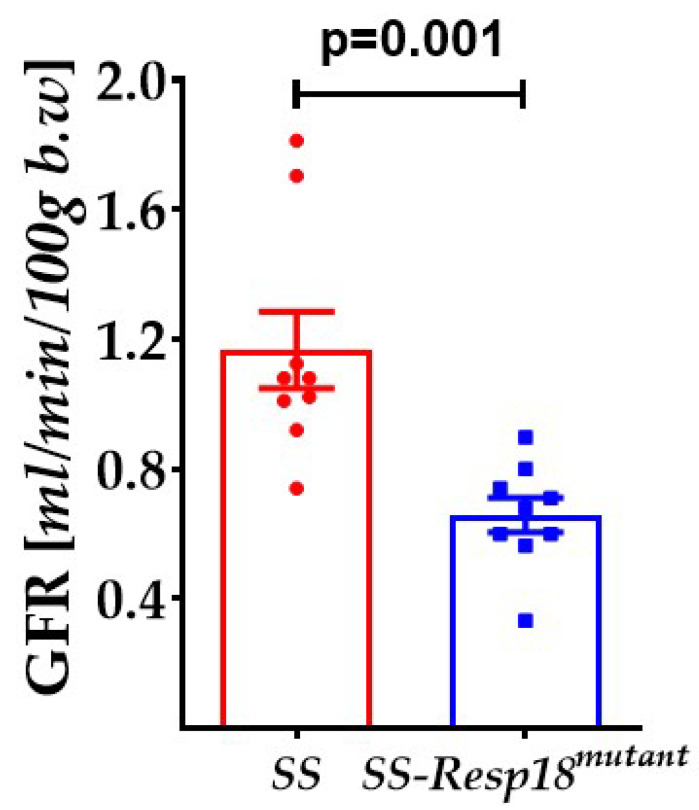
SS-*Resp18^mutant^* rats have decreased GFR: SS and SS-*Resp18^mutant^* rats were injected with FITC-sinistrin. The clearance of FITC-sinistrin in conscious rats was measured via the fluorescence detector NIC-kidney device placed on the rat’s back (*n* = 8). Data are mean ± SEM. *p* = 0.001 vs. SS-*Resp18^mutant^* rats, *t*-test.

Next, we studied the effect of the high-salt diet on GFR in SS and SS-*Resp18^mutant^* rats. With dietary salt causing an increase in vascular resistance, poor myogenic response, and impairment in vascular relaxation, changes in renal hemodynamics and GFR associated with salt-sensitive hypertension may occur [37,38]. Consistent with those reports, the current studies detected lower GFR in conscious SS-*Resp18^mutant^* than conscious SS rats (Figure 2). 

### 3.2. SS-Resp18^mutant^ Rats Have Alteration in the Pressure–Natriuresis Response

**Figure 3:**
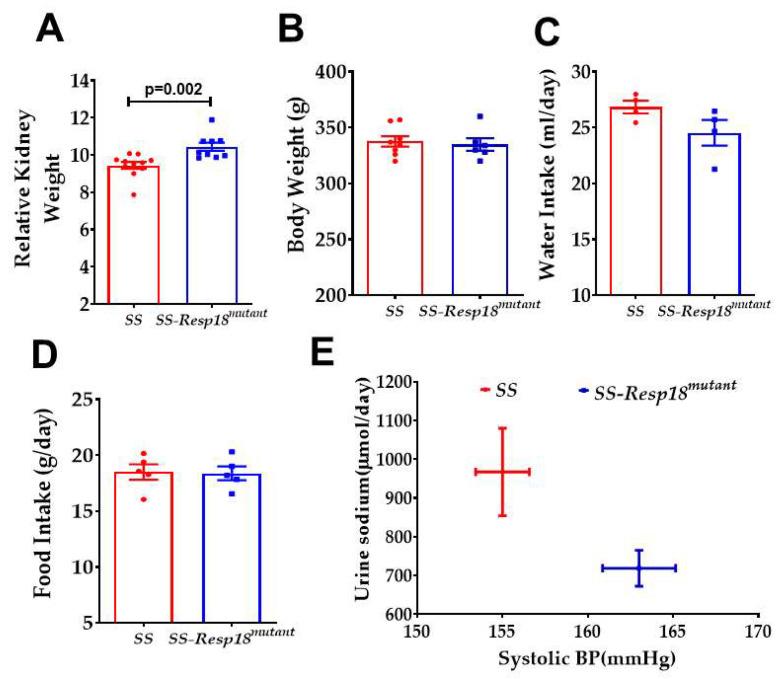
SS-*Resp18^mutant^* rats’ pressure–natriuresis is shifted down and to the right; relative kidney weight is increased in SS-*Resp18^mutant^* rats: (**A**) relative kidney (kidney weight/body weight) (*n* = 9–11), (**B**) body weight (*n* = 9–11) (**C**) water intake (*n* = 4), and (**D**) food intake (*n* = 4) were measured in both SS and SS-*Resp18^mutant^* rats six weeks after high-salt diet. (**E**) Relationship between sodium excretion and blood pressure after six weeks on a high-salt diet. Data are mean ± SEM. *p* = 0.002, vs. SS-*Resp18^mutant^*, *t*-test.

*SS-Resp18^mutant^* rats had an increase in relative kidney weight relative to SS rats (Figure 3A)**,** without significant differences in food intake, water intake, and body weight, in response to a high-salt diet (Figure 3B–D). The kidney plays a pivotal role in the long-term regulation of blood pressure, in part, by the pressure–natriuresis mechanism that connects renal perfusion pressure to the excretion of sodium and water [39]. SS-*Resp18^mutant^* rats exhibited a downward and rightward shift in the relationship between blood pressure and sodium excretion in response to a high-salt diet (Figure 3E), indicating impaired pressure–natriuresis response in these mutant rats.

### 3.3. SS-Resp18^mutant^ Rat Kidneys Exhibit an Increase in Macrophage Infiltration

**Figure 4:**
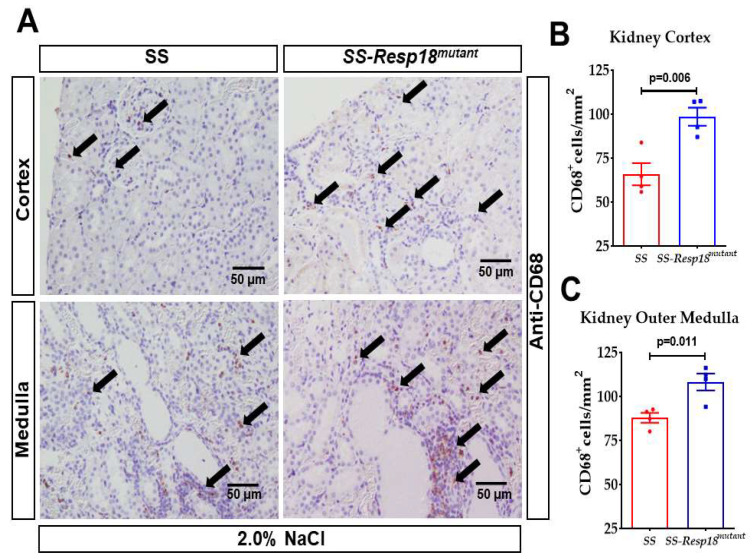
SS-*Resp18^mutant^* rats have increased renal macrophage infiltration: (**A**) Representative images of renal sections probed with CD68 antibody and black arrows indicates positive staining for CD68, (**B**) kidney cortex, (**C**) kidney outer medulla (*n* = 4). Graphs represent the quantification of the percent of the area with CD68^+^ macrophage infiltration. Data are mean ± SEM. *p* = 0.006, *p* = 0.011 vs. SS-*Resp18^mutant^*, *t*-test.

As we have previously shown, SS-*Resp18^mutant^* rats have an increase in renal fibrosis in response to high salt intake [21]. Monocytes/macrophages are involved in the pathogenesis of both experimental and human renal diseases and are implicated in the induction of renal injury and fibrosis [40,41]. In addition, macrophage cell infiltration mediates local injury during the progression of CKD. Consistent with these reports, immunohistochemical analysis showed an increase in CD68+ positive macrophage infiltration in the cortex and outer medulla of SS-*Resp18^mutant^* rat kidneys compared with SS rat kidneys (Figure 4A,B).

### 3.4. Dysregulation of Renal Dopaminergic System in SS-Resp18^mutant^ Rats

**Figure 5:**
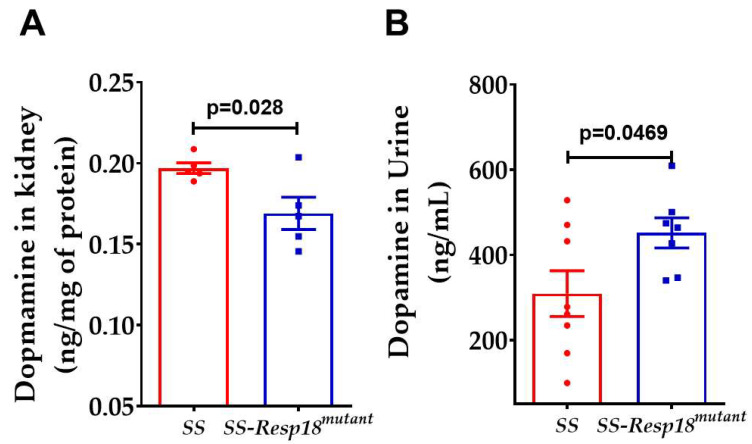
SS-*Resp18^mutant^* rats have increased urinary dopamine: After six weeks of a high-salt diet, dopamine concentration was measured in the kidney (*n* = 5/group) (**A**) and the urine (*n* = 7–8/group) (**B**) of SS and SS-*Resp18^mutant^* rats. Data are mean ± SEM. *p* = 0.028, *p* = 0.0469 vs. SS-*Resp18^mutant^* rats, *t*-test.

With the reported expression of *Resp18* in renal proximal tubule cells, the site of dopamine production in the kidney [20], and with *Resp18* gene expression regulated by dopaminergic drugs [19], it is possible that targeted disruption of *Resp18* interrupts the renal dopaminergic system. To test this hypothesis, we measured intrarenal and urinary dopamine concentrations in a high-salt diet-fed SS and SS-*Resp18^mutant^* rats. Following six weeks of a high-salt diet, dopamine concentrations in the cortical slices of SS-*Resp18^mutant^* rat kidneys were reduced (Figure 5A), but urinary dopamine concentrations were increased in SS-*Resp18^mutant^* compared with SS rats (Figure 5B). The increase in urinary dopamine concentration observed in the high-salt diet fed SS-*Resp18^mutant^* rats implies induction of dopamine synthesis within the kidney in response to a high sodium intake. Renal endogenous dopamine acts as a natriuretic hormone [23,24,25,26,27,42].

**Figure 6:**
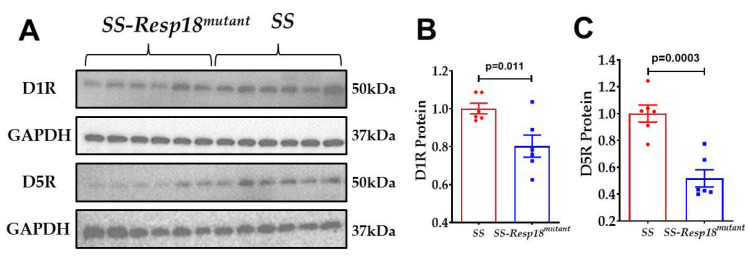
SS-*Resp18^mutant^* rats have decreased renal D1-like receptor protein expression: SS and SS-*Resp18^mutant^* rats were maintained on a high-salt diet for six weeks, and then the rat kidneys were harvested. Kidney protein lysates were immunoblotted for (**A**) D1R and D5R protein in SS and SS-*Resp18^mutant^* rats, and (**B**,**C**) respective expressions were quantified by densitometry (*n* = 6). Data are mean ± SEM. *p* = 0.011, *p* = 0.0003, vs. SS-*Resp18^mutant^* rats, *t*-test.

Dopamine exerts its anti-hypertensive effects, in part by occupation of D_1_-like dopamine receptors, i.e., D1R and D5R [23,24,25,26,27]. Western blot analysis detected a significant reduction in D1R and D5R protein expression in SS-*Resp18^mutant^* rat kidneys (Figure 6).

**Figure 7:**
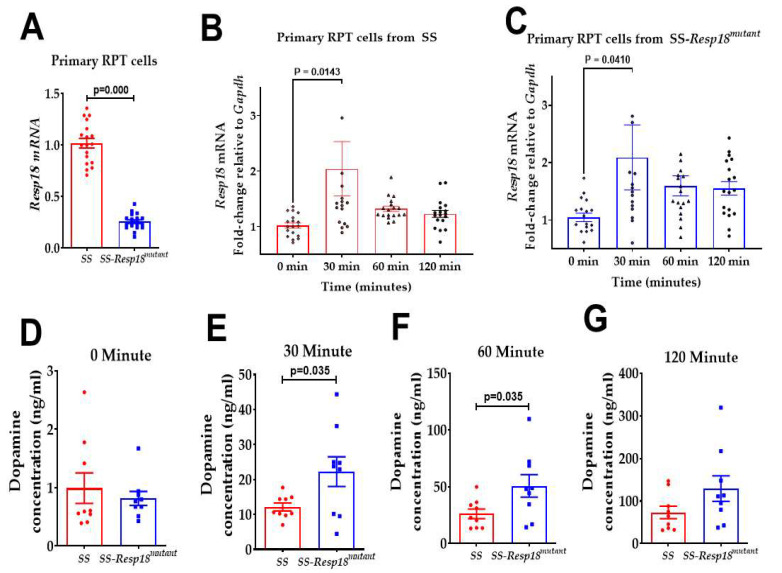
Renal proximal tubule (RPT) cells isolated from the SS-*Resp18^mutant^* rats have increased dopamine production: (**A**) *Resp18* mRNA expression levels in RPT cells from SS and SS-*Resp18^mutant^* rats (*n* = 4). (**B**,**C**) *Resp18* mRNA expression levels were measured in RPT cells treated with L-DOPA (75 μM) in the presence of the monoamine oxidase inhibitor pargyline (10 µM) and the catechol-O-methyltransferase inhibitor tolcapone (1 µM). **(B**) SS rats and (**C**) SS-*Resp18^mutant^* rats (*n* = 8–9). Dopamine levels were measured in RPT cells from SS and SS-*Resp18^mutant^* rats before (**D**) and after the L-DOPA treatment (**E**) 30 min (*n* = 9), (**F**) 60 min (*n* = 9), and (**G**) 120 min (*n* = 9). Data are mean ± SEM. *p* = 0.0143, *p* = 0.0410, vs. 0 min, two-way ANOVA (Sidak test) and *p* = 0.000, *p* = 0.035, vs. SS-*Resp18^mutant^ t*-test.

Restricted D1R availability may limit dopamine action and cause a compensatory increase in renal dopamine production followed by secretion into the tubular lumen, and thus an increase in urinary dopamine (Figure 5B) and a decrease in renal dopamine in SS-*Resp18^mutant^* rats (Figure 5A). To test this hypothesis, we measured *Resp18* gene expression in primary cultures of renal proximal tubule cells from *SS* and SS-*Resp18^mutant^* kidney cortical slices as well as dopamine content at their culture media. At the basal level, *Resp18* expression was significantly lower in renal proximal tubule cells isolated from SS-*Resp18^mutant^* compared with SS rats (Figure 7A). L-DOPA increased *Resp18* gene expression, reaching a peak at 30 min of treatment (Figure 7B,C) and decreasing to the basal level at 60–120 min in SS rat renal proximal tubule cells (Figure 7B). By contrast, *Resp18* expression remained higher than the basal level at 30 to 120 min in renal proximal tubule cells from SS-*Resp18^mutant^* rats (Figure 7C). Moreover, we observed a steady-state increase in dopamine release into the incubation media in renal proximal tubule cells from both control and mutant rats (Figure 7D–G) with higher levels in *Resp18^mutant^* than SS rat renal proximal tubule cells.

## 4. Discussion

We have previously shown that SS-*Resp18^mutant^* rats maintained on a high-salt diet for six weeks displayed a hypertensive phenotype with increased renal fibrosis and urinary protein excretion [21]. The current studies demonstrated that these mutant rats had increased vascular resistance, as shown by reduced response to a vasodilating agent, such as SNP. We also observed that SS-*Resp18^mutant^* rats have a pressure–natriuresis defect, as they exhibited a shift in pressure–natriuresis curve downward and to the right of SS rats, indicating that these mutant rats excrete less sodium even at higher blood pressure than SS rats. However, time-course measurements of renal sodium handling could have provided additional insights on the mutant rats’ pressure–natriuresis response that were potentially missed by the endpoint measurement. Nevertheless, the current studies also showed that these mutant rats have reduced GFR and increased macrophage infiltration in their kidneys. Furthermore, these mutant rats had a decrease in renal dopamine concentration and an increase in urinary dopamine excretion, in parallel with a significant reduction in their renal D1R and D5R protein levels. Together, these studies suggest the dysregulated D_1_-like receptors in SS-*Resp18^mutant^* rat kidneys. Although D_1_-like receptor responses were not further investigated in vivo, the findings in the current study support the hypothesis that targeted disruption of the *Resp18* gene leads to a rise in blood pressure, accompanied by a decrease in GFR and natriuretic function, involving dysregulation of the renal dopaminergic system, relative to that observed in the SS rats [24]. Pressure–natriuresis occurs when sodium excretion is increased secondarily to the increase in blood pressure and renal perfusion pressure [7,43]. A defect in the pressure–natriuresis response can lead to hypertension [7,43]. The current study showed impairment in the pressure–natriuresis response in SS-*Resp18^mutant^* rats, as demonstrated by a significant increase in blood pressure and lower sodium excretion when compared with SS controls. Thus, the decline in GFR in SS-*Resp18^mutant^* relative to SS control rats on the high-salt diet could be part of the impaired pressure–natriuresis response [44].

The slopes of the pressure–natriuresis response in Dahl SS and Dahl salt-resistant rats are similar but that of the former is shifted to the right of the latter following exposure to a high-salt diet [45]. This resetting is not related to renal cortical and papillary blood flow or renal interstitial pressure but rather due to increased renal tubular sodium transport [45]. In the current study, we observed that the pressure–natriuresis response in SS-*Resp18^mutant^* rats was shifted down and to the right of SS rats, which was associated with impaired vasorelaxation response to SNP. Moreover, it has been shown in humans that fenoldopam, a D_1_-like dopamine receptor agonist, relaxes the vascular smooth muscle in vitro [46]. Hence, we contemplate that loss of *Resp18* in SS rats negatively affects the myogenic response primarily through a smooth muscle cell-dependent manner, as evident with the vascular myograph findings. In addition, it is possible that the decrease in GFR observed in the SS-*Resp18^mutant^* rats could be due to vasoconstriction of the afferent arterioles, which may, initially, serve to protect the kidney from hydrostatic pressure damage [47,48].

The increase in perfusion pressure and impaired renal myogenic response in SS-*Resp18^mutant^* rats could have led to renal injury [48,49], as evidenced by the increase in renal fibrosis, urinary protein excretion [21], and macrophage infiltration [50]. The renal inflammation in SS rats, however, may be independent of the increase in blood pressure [51]. Taken together, our findings suggest that the *Resp18* gene is critical in maintaining an appropriate kidney function and blood pressure in an SS rat model for hypertension. *Resp18* is expressed in renal proximal tubule cells [20], where L-DOPA is converted into dopamine, independent of the central nervous system [25,26,52]. *Resp18* gene expression is regulated by dopaminergic agents; the D_2_-like receptor agonist bromocriptine decreases *Resp18 mRNA* levels, whereas the D_2_-like receptor antagonist haloperidol increases *Resp18 mRNA* levels [19]. In the current study, we found that dopamine concentrations were lower in renal cortical slices of SS-*Resp18^mutant^* than in SS rats. However, the urinary dopamine concentrations were higher in the mutant rats, indicating an increase in the secretion of dopamine into their tubular lumens. In normotensive humans and rodents, renal dopamine production is increased in response to high-salt intake [23,24,25,26,27,42]. Dahl SS rats actually have reduced urinary dopamine production with salt loading [53]. Dopamine in the kidney plays a significant role in regulating renal sodium excretion [23,24,25,26,27,53,54,55,56,57]. Dopamine decreases renal tubular sodium reabsorption by inhibiting sodium cotransporters, ion channels, sodium pump, and sodium exchangers, such as NHE3, in renal proximal tubule cells [23,24,25,26,27,53,54,55,56,57]. Dopamine’s anti-hypertensive effects are carried out through the stimulation of the five dopamine receptor subtypes, including D1R and D5R [23,24,25,26,27,53,54,55,56,57]. The present study found reduced expression of D1R and D5R in SS-*Resp18^mutant^* rat kidneys. D1R and D5R are expressed in almost all segments of the nephron, including the proximal tubule, as well as in the tunica media of the arterioles [23,24,25,26,27,53,55,56,58]. Disruption of the *D_5_R (Drd5)* gene in mice causes hypertension that is aggravated by increased salt intake [59]. More interestingly, *D_5_R* deficient mice [59] also exhibit a rightward shift in the pressure–natriuresis response similar to that observed in SS-*Resp18^mutant^* rats. Additionally, the downregulation of D1R has been shown to adversely affect renal function, thus playing a vital role in the pathogenesis of hypertension [60,61]. The inflammation in SS-*Resp18^mutant^* rat kidneys may also be related to dopamine receptors’ dysfunction.

The dopamine release assay in the current study demonstrated an increase in dopamine secretion into the culture medium of renal proximal tubule cells isolated from SS-*Resp18^mutant^*, as compared with SS rats. However, unlike SS renal proximal tubule cells, the expression of *Resp18* remained upregulated 120 min after L-DOPA treatment. By contrast, the *Resp18* expression in SS renal proximal tubule cells peaked at 30 min and fell to basal levels 60 to 120 min post-treatment. This shows a tight negative feedback relationship between *Resp18* gene expression and dopamine production in renal proximal tubule cells. Our findings are also in agreement with published reports on the negative regulation of *Resp18* gene expression by dopamine agonists and its positive regulation by dopamine antagonists [19]. It is well stablished that a correlation exists between the dietary intake of sodium and renal dopamine production/excretion in both humans and laboratory animals [23,24,25,26,27,42,53,54,55,62,63]. Consistent with these reports, dopamine production was persistently greater in the renal proximal tubules from SS-*Resp18^mutant^* rats than SS rats, as reflected by the increase in urinary dopamine excretion and increased levels in the culture media of isolated renal proximal tubule cells. Nevertheless, the natriuresis with salt loading was less in SS-*Resp18^mutant^* than SS rats, suggesting impaired renal dopamine receptors’ function in these rats. Therefore, the hypertensive phenotype observed in SS-*Resp18^mutant^* rats is likely caused by the dysregulated renal dopaminergic system. Further studies are required to extend our current understanding of the role of this novel endocrine protein *Resp18* in renal dopaminergic receptor function and signaling.

## 5. Conclusions

Overall, the current study highlighted the physiological relevance of *Resp18* in regulating blood pressure homeostasis and renal function using a novel global SS-*Resp18^mutant^* rat model maintained on a high-salt diet. The current study showed that a high salt intake increased vascular resistance, decreased GFR, and caused a downward and rightward-shift in the pressure–natriuresis response curve in SS-*Resp18^mutant^* rats*,* relative to SS rat controls. However, the current study is limited by the lack of studies on the time course of the pressure–natriuresis response, which could have provided additional insights on the role of the renal dopaminergic system in the regulation of sodium balance in SS*-Resp18^mutant^* rats. *Resp18* mutation caused dysregulation in the renal dopaminergic system, further unraveling a previously unrecognized physiological role of *Resp18*, an emerging endocrine protein, in regulating blood pressure homeostasis and renal function.

## Data Availability

Not applicable.
